# Immunological and Clinical Impacts of Targeted Intraoperative Radiotherapy in Breast Cancer: A Narrative Review

**DOI:** 10.7759/cureus.94270

**Published:** 2025-10-10

**Authors:** Yastira Ramdas, Pieter W Meyer, Catherine Worsley

**Affiliations:** 1 Radiation Oncology, Corewell Health, Royal Oak, USA; 2 Immunology, National Health Laboratory Service, Pretoria, ZAF; 3 Immunology, University of Pretoria, Pretoria, ZAF

**Keywords:** breast cancer, immunomodulation, intraoperative radiotherapy, tumor microenvironment, wound fluid

## Abstract

Breast cancer (BCa) is the second most diagnosed malignancy among women worldwide and is commonly treated with breast-conserving surgery (BCS) followed by radiotherapy (RT), such as targeted intraoperative radiotherapy (TARGIT-IORT). TARGIT-IORT delivers a single high dose to the tumor bed during surgery, providing local control while minimizing exposure to the surrounding tissues. However, the clinical role of IORT has become highly contested. The adoption of ultrashort external beam schedules eliminates many of its practical advantages, and the 2024 ASTRO guideline no longer recommends IORT outside clinical trials or registries.

This narrative review summarizes evidence on the immunological and biological modulation of the tumor microenvironment (TME) by TARGIT-IORT/IORT, highlighting effects on cytokine profiles and wound healing dynamics. Rather than proposing routine clinical use, the review frames IORT as a translational model to study perioperative immunomodulation in breast cancer.

Analyses of surgical wound fluid, immune biomarkers, and microRNA (miR) expression indicate that TARGIT-IORT/IORT disrupts tumor-supportive wound healing and reshapes local immune signaling. Early trial data suggested non-inferior outcomes compared with external beam radiation, but other studies reported higher recurrence risks. These divergent results, together with the availability of FAST-Forward external beam radiotherapy (EBRT), have limited IORT’s relevance for standard care. Variability in immune findings underscores the need for standardized biomarker assessment protocols and supports positioning IORT as a research platform rather than a clinical replacement.

Future studies should evaluate immune-modulating strategies alongside TARGIT-IORT within prospective trials, refine perioperative biomarker profiling, and integrate these findings with broader immuno-oncology approaches. Although not guideline-endorsed for routine treatment, IORT remains valuable as a translational tool for investigating perioperative tumor-immune interactions in breast cancer.

## Introduction and background

Female breast cancer (BCa) is the second most common cancer worldwide, accounting for a significant proportion of new cancer cases and cancer-related deaths globally [1]. BCa consists of three major subtypes: hormone receptor (HR)-positive BCa (expressed as estrogen receptors (ER) and/or progesterone receptors (PR)), HER-2 amplified BCa, and triple-negative BCa (TNBCa) [2].

Many patients with BCa are eligible for breast-conserving surgery (BCS) with adjuvant systemic therapy instead of total mastectomy [3,4]. Post-BCS, microscopic cancer foci can exist in different quadrants of the residual breast tissue, and most local recurrences occur at or near the original tumor site [3,5-7]. In early-stage BCa, adjuvant radiotherapy (RT) is applied to the tumor bed during BCS. This method underpins the innovation of targeted intraoperative radiotherapy (TARGIT-IORT) [4,5,8,9]. TARGIT-IORT provides a single, high dose of radiation (typically 20 Gy in 15 minutes) directly to the tumor bed during surgery, ensuring precise targeting of the area at the highest risk for recurrence while reducing radiation exposure to healthy tissue [2,3,6,10-13]. Emerging evidence indicates that the benefits of TARGIT-IORT extend beyond direct tumor cell destruction, potentially involving immunological modulation of the tumor microenvironment (TME) (the cellular and molecular milieu that supports or restrains tumor growth), epithelial-mesenchymal transition (EMT) (the process by which epithelial cells acquire invasive, migratory properties), and cancer stem cells (CSCs) (a subpopulation of tumor cells with self-renewal capacity). In addition, the timing of “wound fluid” sampling (e.g., intraoperative and postoperative days 1-3) is critical for understanding alterations in wound healing processes, thereby reducing the likelihood of recurrence [7,14].

The role of intraoperative radiotherapy (IORT) has become increasingly contested. The widespread adoption of ultrashort external beam schedules eliminates many of its logistical advantages. Importantly, IORT is delivered through distinct modalities: low-energy photon IORT (most commonly with the INTRABEAM device) and electron-based IORT [15]. These differ in dose rate, penetration, and depth-dose characteristics, which may in turn influence the magnitude and quality of immunological effects. Moreover, randomized trials have produced divergent long-term recurrence outcomes. The 2024 ASTRO breast cancer guideline advises that IORT should be restricted to use within clinical trials or multi-institutional registries [16].

This narrative review synthesizes contemporary research on the immunological effects of TARGIT-IORT/IORT in breast cancer, with particular emphasis on its influence on TME. The review does not present IORT as a substitute for EBRT but instead evaluates its translational significance. Key findings that illustrate how IORT modulates immune responses in breast cancer are summarized in Table 1. To guide this analysis, we address the following questions. Does IORT alter perioperative inflammatory signaling pathways? Does it reprogram the wound healing response in ways that mitigate tumor-promoting processes? And can the immune modulation induced by IORT provide a biologically plausible rationale for its integration into multimodal breast cancer care? The sections that follow examine evidence from wound fluid biology, immune cell dynamics, microRNA (miR) regulation, and clinical outcomes, including contemporary guideline recommendations.

**Table 1:** Summary of clinical trials conducted and the effects of IORT on the TME IORT: intraoperative radiotherapy, WF: wound fluid, MCF7: Michigan Cancer Foundation-7, MSC: mesenchymal stromal cell, MDA-MB-231: MD Anderson-metastatic breast cancer cell line 231, CSC: cancer stem cell, EMT: epithelial-to-mesenchymal transition, CTL: cytotoxic T lymphocyte, NK: natural killer (cell), Treg: regulatory T cell, IL-1β: interleukin-1 beta, IL-6: interleukin-6, IL-7: interleukin-7, IL-8: interleukin-8, IL-10: interleukin-10, IL-13: interleukin-13, TNF-α: tumor necrosis factor alpha, TNF-β: tumor necrosis factor beta, EGF: epidermal growth factor, VEGF: vascular endothelial growth factor, VEGF-R3: vascular endothelial growth factor receptor 3, FGF: fibroblast growth factor, HGF: hepatocyte growth factor, PDGF-BB: platelet-derived growth factor-BB, IGFBP-6: insulin-like growth factor binding protein 6, GRO-α: growth-regulated oncogene alpha, MIP-1d: macrophage inflammatory protein-1 delta, RANTES: regulated on activation, normal T cell expressed and secreted, DLL4: delta-like ligand 4, miR: microRNA, EGFR: epidermal growth factor receptor, sTNFR-I: soluble tumor necrosis factor receptor I, sTNFR-II: soluble tumor necrosis factor receptor II, uPAR: urokinase plasminogen activator receptor, Tie-1: tyrosine kinase with immunoglobulin and EGF homology domains 1, Tie-2: tyrosine kinase with immunoglobulin and EGF homology domains 2, OSM: oncostatin M, MCP-1: monocyte chemoattractant protein-1, MCP-2: monocyte chemoattractant protein-2, XDH: xanthine dehydrogenase, CDH3: cadherin 3, SOCS3: suppressor of cytokine signaling 3, GABBR2: gamma-aminobutyric acid type B receptor subunit 2, GBP2: guanylate binding protein 2, SLC12A2: solute carrier family 12 member 2, COLCA2: colorectal cancer associated 2, CMYA5: cardiomyopathy associated 5, CDK1: cyclin-dependent kinase 1, CCNA1: cyclin A1, CRISP3: cysteine-rich secretory protein 3, LEC: lymphatic endothelial cell

Author, year, and location	Key findings	Upregulated	Downregulated
Orozco et al. (2024), USA [17]	IORT altered tumor microenvironment, showing squamous metaplasia with atypia (63.6%, p=0.004) and immune pathway enrichment (T-cell activation, inflammatory response); N=22	875 upregulated gene expressions, dendritic cells, monocytes, γδ T cells, and immune score	787 downregulated gene expression
Torabinejad et al. (2023), Iran [18]	WF from IORT+ patients increased MCF-7 growth (p<0.01) but reduced migration compared to IORT- (p<0.02); 37 patients: 18 BCS alone and 19 BCS+IORT	Mean value of light absorbance for groups were reported rather than individual markers	
Nafissi et al. (2022), Iran [19]	Significant differences were observed between pre- and post-surgery serum levels; the IORT group had reduced death and recurrence rates; N=360	EGF (IORT), VEGF (IORT)	DLL4
Pan et al. (2022), China [20]	IORT significantly changed miRNA expression, with miR-4485-3p, miR-215-5p, and miR-2276-5p upregulated, and miR-6886-5p and miR-500-y downregulated	hsa-miR-4485-3p, hsa-miR-215-5p, and hsa-miR-2276-5p	hsa-miR-6886-5p, miR-500-y, and miR-6544-x
Wuhrer et al. (2021), Germany [21]	WF from IORT-treated patients inhibited MSC proliferation, wound healing, and migration, but had no effect on MDA-MB-231 cells; N=42	Leptin	GRO-α, IL-1β, and OSM
Kulcenty et al. (2020), Poland [22]	IORT modified WF by enhancing DNA repair pathways, reducing IL-6 and JAK/STAT3 activation, and altering inflammatory response	XDH, IL-22, IL22RA2, CDH3, SOCS3, GABBR2, GBP2, SLC12A2, COLCA2, CMYA5, CDK1	A1 (CCNA1)
Kulcenty et al. (2019), Poland [23]	WF promotes CSC traits and EMT in breast cancer cells, but this effect is reduced after IORT; the radiation-induced bystander effect contributes to these changes in wound fluid properties; N=43: 22 BCS+IORT and 21 BCS	CD44+/CD24- (CSC marker expression), EMT-associated traits	CSC phenotype expression (especially in MCF7 cells), EMT features
Kulcenty et al. (2019), Poland [24]	Higher expression of antitumor cytokines was seen in luminal A BCa after IORT, with reduced tumor-facilitating cytokines	CTACK, HGF cytokine, G-CSF, HGF, IL-1β, and protein 3 (CRISP3), and KIAA1324 TNF-α	IL-7, IL-8, IL-13, MIF, and TNF-β
Linares et al. (2019), Spain [25]	IORT increased cytotoxic T cells (CTLs) and NK cells, whereas EBRT reduced them, impacting immune response; N=45	Cytotoxic T cells, NK cells, and monocytes stable (CTL)	Treg (luminal A) and granulocytes
Kulcenty et al. (2018), Poland [26]	WF from IORT patients induced apoptosis in MCF7 cells, while WF from non-IORT patients had no effect	CD44+/CD24−	-
Zaleska et al. (2017), Poland [27]	WFs from BCS-only patients led to higher upregulation of miR-21, miR-155, and miR-221 in SK-BR-3 (HER2-positive) cells compared to WFs from BCS+IORT patients; both WFs and RT-WF downregulated miRNAs in basal/epithelial and luminal BCa subtypes	-	miR-21, miR-221, and miR- 155
Scherer et al. (2016), Germany [28]	IORT had no impact on TGF-β1 or HA levels, but TGF-β inhibited LEC proliferation	No effect on TGF-β1, HA, or sHA	
Fabris et al. (2016), Italy [29]	IORT induced miR-223 expression in peritumoral breast tissue; decreased EGF-EGFR signaling disrupted the post-surgical wound healing stimulation loop, potentially reducing recurrence	miR-223	EGF (IORT)
Veldwijk et al. (2015), Germany [30]	A non-significant trend toward reduced proliferation was observed in the MTT assay with 1% IORT-treated WF (p=0.07), but not at 3% (p=0.16); N=30: 12 IORT and 18 non-IORT	No effect on proliferation in BCa MCF7 cell lines	
Belletti et al. (2008), Italy [14]	WF from non-IORT patients stimulated BCa cell proliferation, while WF from TARGIT-IORT patients reduced tumor progression	AgRP, EGFR, FAS/TNFRSF6, FGF-4, G-CSF, IGFBP-6, IL-13 (IORT), IL-4, IL-5, Mip-1d	Angiogenin, Flt-3 ligand, IL-10, IL-6, IL-7, IL-8, leptin, MCP-1, MCP-2, RANTES, PDGF-BB, GRO, HGF (IORT), MIP-1a (IOERT), sTNFR-II, sTNFR-I, uPAR, VEGF-R3 (IORT), Tie-1, Tie-2

## Review

Methods

This review focuses on early-stage female BCas treated with IORT, categorized as low risk, with no restrictions applied to immunological research domains. During the review process, abstracts and full-text articles were screened to ensure that studies included the appropriate patient population. Studies restricted exclusively to high-risk BCa were excluded. Review papers were excluded in favor of original clinical trials, with follow-up reports incorporated only when they contributed additional immunological data.

A systematic search was conducted across PubMed, Web of Science, Scopus, Embase, Directory of Open Access Journals (DOAJ), ClinicalTrials.gov, National Institute for Health Research (NIHR), SpringerLink, Wiley, and Elsevier from inception to February 2025. Both standardized MeSH terms and free-text keywords (such as IORT, TARGIT, intraoperative radiotherapy, and breast cancer) were used to identify relevant English-language studies.

Inclusion criteria were as follows: (1) partial or whole breast irradiation (PBI) techniques including TARGIT-IORT/IORT, (2) early-stage BCa, (3) English-language publication, (4) systematic reviews, meta-analyses, clinical trials, cohort studies, case-control studies, or case series, (5) immunological outcomes, and (6) defined outcome indicators. Exclusion criteria were as follows: (1) incomplete or unpublished studies, (2) animal studies, and (3) advanced-stage cancers. As this was a systematic review, ethics or institutional review board approval was not required.

Pre-selected data were extracted to standardize reporting across studies, ensuring consistent capture of author, year, location, study objective, hypothesis, study design, methodology, immunological pathways, key findings, clinical implications, and directional regulation of immune markers. Due to substantial heterogeneity in study design, immunological endpoints, and outcome reporting, a formal meta-analysis was not feasible.

Effects of IORT on the TME

Although IORT is no longer recommended for routine treatment, translational studies remain informative for understanding perioperative immune responses. Analyses of re-excised tissue, wound fluid, and immune markers consistently show that IORT alters cytokine landscapes, immune cell composition, and microRNA expression compared with surgery alone or EBRT. These findings reinforce IORT’s value as a model for studying how localized perioperative radiation modifies the TME.

To understand how IORT shapes the TME at the molecular level, researchers have analyzed re-excised tissue following BCS with margin revision. Among patients who received IORT (n=22), gene expression profiling revealed a marked shift in the local immune landscape. Pathways associated with inflammatory signaling, granulocyte activation, and T-cell responses were significantly upregulated (p<0.001), indicating an activated immunological state. Strikingly, these changes were not confined to the tumor bed; adjacent normal tissue also showed activation of intrinsic apoptotic and programmed cell death pathways, suggesting that IORT may initiate broader immunological reprogramming beyond the site of irradiation [17].

These molecular shifts are reflected in the changes in the cytokine landscape. A study that evaluated early immune dynamics in 42 patients (21 per group) found that IORT modulated cytokine expression within 24 hours of treatment. While markers of apoptosis remained largely stable, notable differences were observed in inflammatory mediators. Interleukin-1 beta (IL-1β) and growth-regulated oncogene-alpha (GRO-α) levels declined (1.8-fold and 0.6-fold, respectively), although the latter did not reach statistical significance, and leptin levels increased by 1.7-fold (p<0.05). Oncostatin-M was also reduced. In addition to these soluble markers, IORT was associated with a reduction in mesenchymal stromal cells, indicating a potential impact on stromal remodeling [21].

Larger-scale data extend this perspective. In a cohort of 360 patients, Nafissi et al. compared serum biomarker changes in patients who received BCS plus EBRT versus those who received BCS with IORT [19]. Among those treated with IORT, elevated levels of epidermal growth factor (EGF) (p<0.01) and vascular endothelial growth factor (VEGF) (p<0.05) were observed postoperatively, alongside a significant reduction in delta-like ligand 4 (DLL4) (p<0.05). No changes were observed in transforming growth factor beta (TGF-β) or fibroblast growth factor (FGF) levels; however, the angiogenic profile associated with IORT differed meaningfully from that associated with EBRT, again suggesting a distinct biological imprint left by intraoperative exposure [19].

TARGIT-IORT/IORT induces immediate, localized biological changes that extend beyond cytotoxicity effects. Its influence on immune signaling, cytokine dynamics, and stromal composition supports the idea that this form of radiotherapy serves not only as a therapeutic tool but also as a biological catalyst, capable of transforming the microenvironment in ways that may influence tumor recurrence, immune surveillance, and treatment synergy.

Taken together, wound fluid studies suggest that IORT shifts the postoperative environment away from a pro-tumorigenic, EMT-promoting milieu and toward one enriched for apoptotic and immune-stimulatory signaling. These biological signals may not justify routine clinical adoption of IORT given current recurrence concerns, but they provide a framework for designing perioperative immunomodulatory strategies.

IORT and wound fluid (WF) composition

Molecular crosstalk within surgical WF offers a unique window into how TARGIT-IORT/IORT reshapes the TME. Following BCS, the composition of this fluid differs depending on whether TARGIT-IORT/IORT is delivered, with signals suggesting dampening of pro-tumorigenic pathways and enhancement of immune-mediated and apoptotic responses. While these translational findings are consistent across several studies, their clinical application is uncertain, as the role of IORT in breast cancer has become highly contested. Thus, the value of WF studies is primarily in highlighting biological insights rather than justifying routine use. Table 2 provides a concise summary of the studies discussed [31].

**Table 2:** IORT affecting TME clinical trial summary WF: wound fluid, BCS: breast-conserving surgery, IORT: intraoperative radiotherapy, EMT: epithelial-to-mesenchymal transition, CSC: cancer stem cell, G-CSF: granulocyte colony-stimulating factor, IL-1β: interleukin-1 beta, TNF: tumor necrosis factor, TRIAL: TNF-Related Apoptosis-Inducing Ligand (also abbreviated as TRAIL), CASP10: caspase-10, IL-6: interleukin-6, IL-10: interleukin-10, VEGF-R3: vascular endothelial growth factor receptor 3, GRO: growth-regulated oncogene, IL-13: interleukin-13, CTL: cytotoxic T lymphocyte, NK: natural killer (cell), TREG: regulatory T cell, miR-21: microRNA-21, miR-221: microRNA-221

Study	Sample size/design	Main findings	Notable biomarkers/pathways
Kulcenty et al. (2019) [23]	n=~40; WF analyzed post-BCS+IORT	IORT enriched immune/apoptotic signaling; reduced EMT and CSC traits	G-CSF, IL-1β, TNF, TRIAL, CASP10
Belletti et al. (2008) [14]	n=45; pre-/postoperative WF compared in cell cultures	↓ IL-6, IL-10, VEGF-R3, GRO; ↑ G-CSF, IL-13	174 analytes; pro-/anti-inflammatory cytokines
Linares et al. (2019) [25]	Immune phenotyping in blood	↑ CTLs and NK cells (IORT); ↑ granulocytes; no change in TREGs	Flow cytometry markers
Zaleska et al. (2017) [27]	WF from HER2+ patients	↑ miR-21, miR-221 in BCS-only WF versus IORT WF	Oncogenic microRNAs

In early-stage BCa, they observed that WF collected after BCS with IORT displayed elevated levels of cytokines, such as granulocyte colony-stimulating factor (G-CSF), hepatocyte growth factor (HGF), and interleukin-1 beta (IL-1β) in luminal A tumors and induced distinct gene expression signatures in luminal B subtypes [22-24,26]. When applied to MCF-7 cells, WF activated pathways associated with cell cycle regulation, DNA repair, and oxidative phosphorylation, and prominently upregulated markers of extrinsic apoptosis, including tumor necrosis factor (TNF), NF-related apoptosis-inducing ligand (TRIAL), and caspase-10 (CASP10). In contrast, WF from patients undergoing surgery alone promoted cancer stem cell traits, epithelial-mesenchymal transition (EMT), and migratory behavior effects substantially attenuated by IORT. These findings suggest that IORT actively transforms the postoperative TME, shifting it from a wound healing milieu to one that is more hostile to residual tumor cells.

This antitumor signature was also found by Belletti et al., where the proliferative and migratory influence of WF from patients treated with BCS alone versus those treated with TARGIT-IORT were compared [14]. They reported broad suppression of angiogenic and inflammatory mediators, including interleukin-6 (IL-6), interleukin-10 (IL-10), vascular endothelial growth factor receptor 3 (VEGF-R3), growth-regulated oncogene (GRO), hepatocyte growth factor (HGF), and monocyte chemoattractant protein-1 (MCP-1), following IORT, while a contrasting upregulation of immune stimulants, such as G-CSF, FGF-4, and IL-13, suggested a reparative yet immune-alert phenotype.

Beyond the secretome, immune cell phenotyping further underscores IORT’s modulatory effects. Linares et al. observed that cytotoxic T lymphocyte (CTL) frequencies increased in patients receiving IORT, with or without subsequent EBRT, while natural killer (NK) cells increased specifically in the IORT-only group. Interestingly, regulatory T cells (Tregs) show no statistical difference, whereas the granulocyte levels increased across all cohorts [25].

At the level of non-coding RNA, investigation by Zaleska et al. [27] into HER2-positive BCa samples found that WF from BCS-only patients induced higher expression of oncogenic microRNAs, miR-21 and miR-221, compared to WF from IORT-treated patients [20]. This difference suggests a role for IORT in mitigating microRNA-driven tumor progression.

TARGIT-IORT/IORT modifies the biochemical and cellular profiles of WF in ways that may inhibit residual cancer cell survival and invasion. While the magnitude of these effects may depend on the timing, tumor subtype, and in vitro modeling conditions, the direction of impact remains consistently antitumorigenic.

Long-term clinical outcomes of IORT

Over the past decade, long-term clinical data have clarified the potential and the limits of IORT. The pivotal TARGIT-A trial of photon-based IORT (INTRABEAM) provided initial reassurance: at five years’ median follow-up, local recurrence was 3.3% (95% confidence interval (CI): 2.1-5.1) in the TARGIT-IORT arm compared with 1.3% (95% CI: 0.7-2.5) in the EBRT arm (p=0.042) [3,6,32]. As the follow-up matured, these findings remained steady. At a median follow-up of nine years, there were no significant differences between the two arms in local control, distant metastasis, breast preservation, or BCa-specific survival. Notably, all-cause mortality was significantly lower among patients receiving TARGIT-IORT, with a hazard ratio of 0.59 (95% CI: 0.40-0.86, p=0.005) [3,6]. This survival advantage appeared to stem largely from a reduction in non-BCa deaths, which declined from 7.5% to 4% over a 10-year span, a 41% relative risk reduction (p<0.05) [32-35]. 

Importantly, these findings are not confined to trial settings. A large single-institutional series involving 814 patients who received TARGIT-IORT during BCS reported consistent outcomes in terms of disease control and survival, reinforcing the generalizability of the TARGIT-A results [33]. With a median follow-up of 72 months, this cohort confirmed that the real-world application of the TARGIT approach can achieve outcomes on par with those seen in controlled trials, as summarized in Table 3.

**Table 3:** TARGIT-IORT summary data TARGIT-A: Targeted Intraoperative Radiotherapy Trial A, EBRT: external beam radiation therapy, CI: confidence interval, HR: hazard ratio, BCa: breast cancer, QoL: quality of life, TGF-β: transforming growth factor beta

Domain	Finding	Reference
Oncologic efficacy	TARGIT-A: non-inferior to EBRT in local recurrence (1.16% difference; upper CI: 1.99%), no difference in local/distant control; lower all-cause mortality (HR: 0.59, p=0.005), 41% reduction in non-BCa deaths (7.5% → 4%)	Vaidya et al. (2021) [6]
	Consistent survival and disease control outcomes	Vaidya et al. (2010) [4]
Access/logistics	Single-dose therapy reduces costs and travel	Coombs et al. (2016) [36]
	Enables treatment in patients with cognitive or movement disorders	Bhimani et al. (2024) [37]
QoL/patient experience	Better cosmetic outcomes and QoL over five years	Corica et al. (2016) [38]
Toxicity and biomarker insight	TGF-β linked to post-radiation fibrosis and scarring	Yang et al. (2023) [39]

These results contrast with the ELIOT trial, which evaluated electron-based IORT in Milan. In that study, local recurrence rates were significantly higher in the IORT arm than with EBRT, underscoring how differences in energy, dose distribution, and delivery technique may translate into divergent oncologic outcomes [15].

In parallel, the adoption of ultrahypofractionated whole-breast radiotherapy has reshaped the comparative landscape. The FAST-Forward trial demonstrated that 26 Gy in five fractions over one week was non-inferior to the standard 40 Gy in 15 fractions, with similar local relapse rates and acceptable normal tissue effects at five years [15,40].

Early randomized evidence suggested non-inferior outcomes compared to EBRT, but subsequent long-term follow-up and divergent trial results have raised concerns about higher recurrence in some cohorts. Although signals of reduced non-cancer mortality remain intriguing, these findings have not been consistently reproduced. In parallel, the widespread adoption of the five-fraction FAST-Forward EBRT schedule has removed many of IORT’s practical advantages [15,40]. The cumulative evidence positions IORT not as a routine alternative to EBRT, but as an investigational modality whose long-term biological signals may inform survivorship research [16].

Advantages of TARGIT-IORT

**Figure 1:**
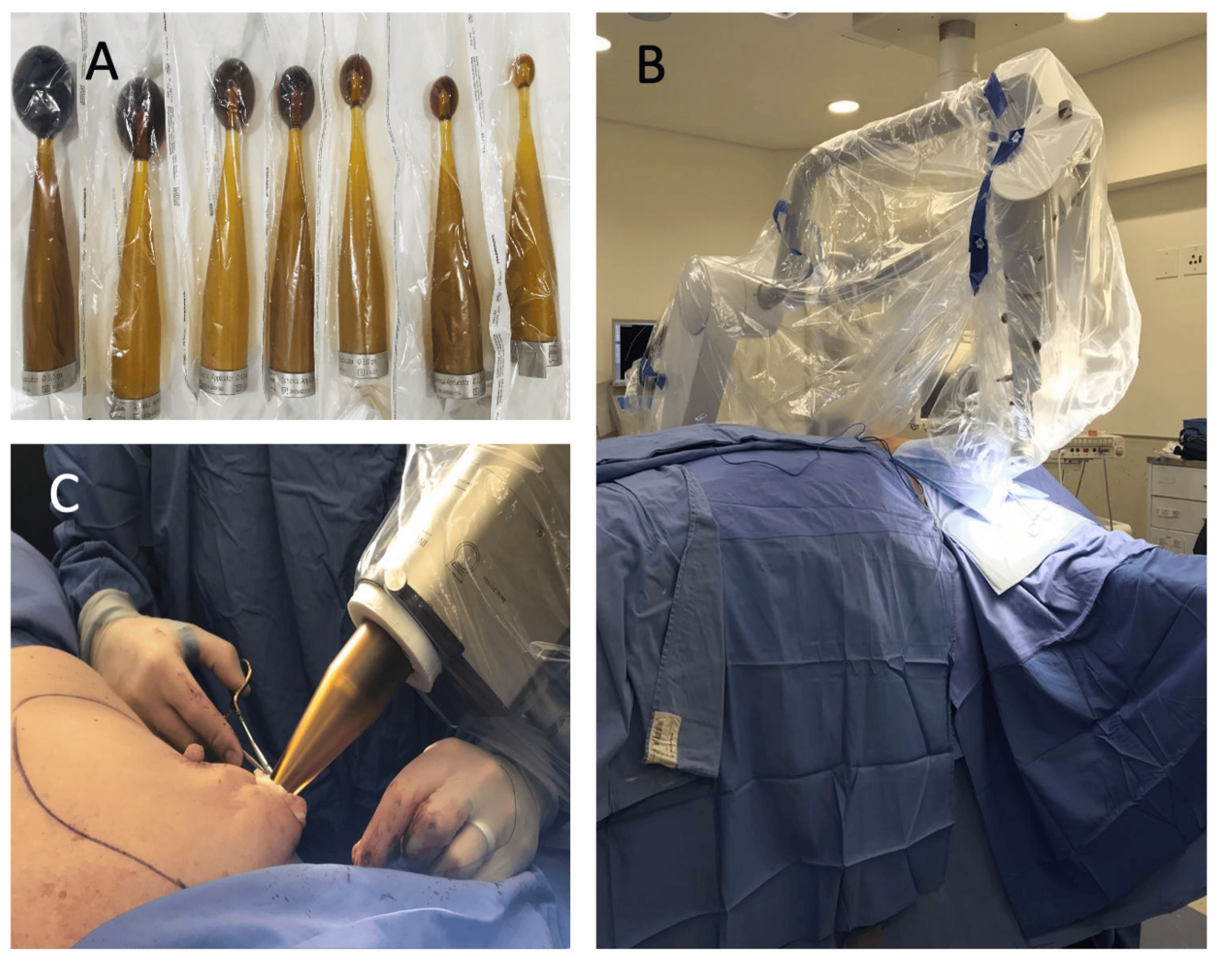
TARGIT-IORT procedure and applicators (A) Various applicator sizes, (B) Carl Zeiss 50kV INTRABEAM administering TARGIT-IORT for a female patient, (C) close-up of plastic surgeon suturing around the applicator. TARGIT-IORT: targeted intraoperative radiotherapy This is an original image with patient consent for images to be published obtained.

The benefits of TARGIT-IORT were originally framed as extending beyond oncologic outcomes, including convenience, cost reduction, and patient-centered delivery at the time of surgery (Table 3, Figure 1). However, the introduction of ultrashort five-fraction EBRT (FAST-Forward) has eliminated many of these practical advantages, as it provides similar logistical ease with stronger long-term evidence. Thus, while IORT continues to offer a unique single-fraction intraoperative option in select clinical or logistical situations, its broader application is limited, and its primary value now lies in translational and investigational research [31,36,39-41]. This streamlined approach not only improves convenience but also opens the door for patient populations who may otherwise struggle to complete protracted treatment schedules.

For instance, patients with cognitive impairments, psychiatric illnesses, or neuromuscular disorders, such as Parkinson’s disease, often find daily radiation intolerable or impractical. In such cases, TARGIT-IORT can be a lifeline. Bhimani et al. highlighted its utility in such contexts, illustrating how the single-fraction delivery expands access to curative treatment for patients who are too often excluded from standard care pathways [37].

Patient-reported outcomes further reinforce the advantages of this method. A longitudinal study by Corica et al. followed 126 patients across three centers over a five-year period and found that those treated with TARGIT-IORT reported better cosmetic satisfaction and overall quality of life (QoL) than their counterparts who received EBRT [38]. These findings suggest that the benefits of TARGIT-IORT are not only practical but also deeply personal, shaping how patients feel, function, and perceive their treatment journey.

An underexplored area in QoL assessments is the use of immune biomarkers to predict likely patient outcomes, particularly those involved in complication pathways, such as TGF-β in radiation-induced fibrosis [28]. TGF-β expression is associated with radiation-induced fibrosis and serves as a key regulator of tissue remodeling after inflammation. It promotes the production of ECM components and stimulates mesenchymal cell proliferation, migration, and accumulation [42,43]. Although essential for wound healing, prolonged activation of this pathway can contribute to fibrosis, resulting in excessive tissue scarring and dysfunction. Measuring these and other biomarkers could provide valuable insights into the biological impact of treatment-related side effects and help predict long-term functional and structural tissue changes, thereby impacting QoL. Incorporating such biomarkers into routine care may eventually help clinicians move beyond a one-size-fits-all approach, optimizing outcomes not only by tumor control but also by preserving long-term tissue integrity, function, and patient well-being.

TME modulation and molecular effects

In the absence of TARGIT-IORT, experimental and clinical studies have reported that patients undergoing BCS may exhibit accelerated tumor regrowth by stimulating stromal cells to produce growth factors during wound healing [14]. While TARGIT-IORT reduces local recurrence by promoting direct tumor cytotoxicity, it may also modulate the peri-tumoral microenvironment in part by altering the wound healing response triggered by surgery [12,14]. In vitro experiments and clinical observations indicated that WF from patients treated with TARGIT-IORT were unable to activate key tumor-promoting pathways, specifically p70S6K and STAT3, compared to those patients who only underwent surgery; however, other signaling pathways, such as AKT and ERK, remained largely unaffected [12]. TARGIT-IORT not only sterilizes the tumor bed but also modulates the wound response, suggesting potential synergy with targeted inhibitors of pathways such as p70S6K and STAT3. While these hypotheses are scientifically compelling, they remain translational and not directly clinically actionable. Given that IORT is no longer guideline-endorsed outside trials, such combinations should be tested exclusively in prospective research protocols. The biological rationale remains strong, but clinical application is currently speculative [12,14,31]. The imbalance in cytokine levels could lead to a distinct immune response within the TME, potentially strengthening the antitumor effects of IORT by modifying immune signaling pathways and enhancing the body’s ability to target residual cancer cells [12].

Controversies and limitations in current evidence

While preclinical and translational literature highlight immunomodulatory effects of IORT, significant inconsistencies in study outcomes and divergent trial results limit generalizability. These clinical uncertainties, coupled with competing ultrashort EBRT schedules, have shifted IORT into a purely investigational space. Variations in wound fluid sampling, assay techniques, and equipment further complicate reproducibility, underscoring the urgent need for methodological standardization if IORT is to remain valuable as a translational research platform.

The results of the study by Torabinejad et al. [18] suggested that IORT post-BCS increased BCa cell growth in contrast to the myriad of studies [12,14,21]. The authors acknowledged this contradiction and attributed deviations from the norm to the collection of blood at 18 hours instead of the typical 24-48 hours and to the potential contamination of WF samples with blood components [18]. Despite these anomalies, the authors affirmed the well-established clinical advantages of IORT and the extensive body of research supporting its use. This highlights the need to standardize immune marker testing and address the complexities and transience within the TME. Veldwijk et al. found no statistically significant reduction in BCa proliferation on MCF-7 lines for patients treated with IORT [30]. However, the authors used multiple WF-to-medium concentrations and observed that only the 100% WF concentration reduced BCa cell proliferation [30]. They did not investigate the impact of TME and cytokine levels between the IORT+BCS and BCS-only groups, which could have an effect [30]. Furthermore, alterations in the composition of immunological markers WF post-RT that varied across studies are likely due to differences in IORT equipment, techniques, and radiation doses [17,30,39]. These methodological inconsistencies complicate comparisons across studies and hinder the ability to draw definitive conclusions regarding the immunological impact of IORT.

Taken together, these limitations highlight a broader issue in the field: the need for methodological standardization. Differences in WF collection timing, processing techniques (e.g., centrifugation and storage conditions), and assay sensitivity may significantly affect experimental results and reduce reproducibility. Without standardized approaches to immune marker analysis and functional assays, the translational significance of preclinical findings remains difficult to determine.

Future studies should address these challenges by implementing consistent protocols for sample handling, applying multi-omic profiling to characterize the TME, and correlating immunological changes with clinical endpoints. Bridging these gaps is critical for refining the mechanistic understanding of TARGIT-IORT/IORT and optimizing its integration into multimodal BCa care.

Emerging biomarkers and predictive tools

MicroRNAs (miRs) are small, non-coding RNA molecules that post-transcriptionally regulate gene expression. Their expression is tissue-specific, with different tissues producing distinct sets of miRs [29]. MiRs play a crucial role in controlling the availability of morphogens and growth factors and are involved in the interactions between tumor cells and the surrounding environment [29,44]. IORT was shown to induce the expression of miR-223 in the tissue surrounding the tumor, which suppresses the expression of EGF, which is responsible for tumor cell growth [29]. Normally, after surgery, the EGF-EGFR pathway creates a feedback loop that promotes tumor cell survival and potential recurrence. Additionally, mouse models have shown that both RT-induced miR-223 expression and perioperative EGFR inhibition effectively prevent BCa cell growth and reduce recurrence rates [29]. This suggests that targeting perioperative pathways in conjunction with IORT may inform biomarker-driven strategies in the future. Importantly, such approaches should be pursued only within the structure of prospective trials, consistent with current ASTRO guidance [16].

## Conclusions

Overall, TARGIT-IORT/IORT should no longer be presented as a standard-of-care alternative to EBRT. Current evidence and ASTRO 2024 guidelines restrict its use to clinical trials or registries, reflecting concerns about recurrence risk and the availability of validated five-fraction external beam regimens. Nonetheless, IORT continues to offer unique translational insights into perioperative immunology, wound healing, and biomarker discovery. Future research should focus on integrating IORT-based immunological findings with systemic immunotherapies, refining biomarker-guided patient selection, and designing trials that leverage perioperative biology to improve outcomes in breast cancer.

## References

[1] Bray F, Laversanne M, Sung H, Ferlay J, Siegel RL, Soerjomataram I, Jemal A (2024). Global cancer statistics 2022: GLOBOCAN estimates of incidence and mortality worldwide for 36 cancers in 185 countries. CA Cancer J Clin.

[2] Higgins MJ, Baselga J (2011). Targeted therapies for breast cancer. J Clin Invest.

[3] Vaidya JS, Bulsara M, Baum M, Tobias JS (2021). Single-dose intraoperative radiotherapy during lumpectomy for breast cancer: an innovative patient-centred treatment. Br J Cancer.

[4] Vaidya JS, Joseph DJ, Tobias JS (2010). Targeted intraoperative radiotherapy versus whole breast radiotherapy for breast cancer (TARGIT-A trial): an international, prospective, randomised, non-inferiority phase 3 trial. Lancet.

[5] Vaidya JS, Vyas JJ, Chinoy RF, Merchant N, Sharma OP, Mittra I (1996). Multicentricity of breast cancer: whole-organ analysis and clinical implications. Br J Cancer.

[6] Vaidya JS, Bulsara M, Baum M (2020). Long term survival and local control outcomes from single dose targeted intraoperative radiotherapy during lumpectomy (TARGIT-IORT) for early breast cancer: TARGIT-A randomised clinical trial. BMJ.

[7] Vaidya JS, Bulsara M, Baum M (2021). New clinical and biological insights from the international TARGIT-A randomized trial of targeted intraoperative radiotherapy during lumpectomy for breast cancer. Br J Cancer.

[8] Vaidya JS (2001). A Novel Approach for Local Treatment of Breast Cancer. https://www.ucl.ac.uk/~rmhkjsv/papers/2002PhDThesisEbookNP.pdf.

[9] Vaidya JS, Baum M, Tobias JS, Morgan S, D'Souza D (2002). The novel technique of delivering targeted intraoperative radiotherapy (Targit) for early breast cancer. Eur J Surg Oncol.

[10] Vaidya JS, Bulsara M, Wenz F (2016). Reduced mortality with partial-breast irradiation for early breast cancer: a meta-analysis of randomized trials. Int J Radiat Oncol Biol Phys.

[11] Vaidya JS, Bulsara M, Saunders C (2020). Effect of delayed targeted intraoperative radiotherapy vs whole-breast radiotherapy on local recurrence and survival: long-term results from the TARGIT-a randomized clinical trial in early breast cancer. JAMA Oncol.

[12] Massarut S, Belletti B, Segatto I, Piccoli E, Baldassarre G (2015). Wound response after intraoperative radiotherapy. Transl Cancer Res.

[13] Ramdas Y, Benn CA, van Heerden M (2020). First intraoperative radiation therapy center in Africa: first 2 years in operation, including COVID-19 experiences. JCO Glob Oncol.

[14] Belletti B, Vaidya JS, D'Andrea S (2008). Targeted intraoperative radiotherapy impairs the stimulation of breast cancer cell proliferation and invasion caused by surgical wounding. Clin Cancer Res.

[15] Veronesi U, Orecchia R, Maisonneuve P (2013). Intraoperative radiotherapy versus external radiotherapy for early breast cancer (ELIOT): a randomised controlled equivalence trial. Lancet Oncol.

[16] Shaitelman SF, Anderson BM, Arthur DW (2024). Partial breast irradiation for patients with early-stage invasive breast cancer or ductal carcinoma in situ: an ASTRO clinical practice guideline. Pract Radiat Oncol 2024;14:112-132. Pract Radiat Oncol.

[17] Orozco JI, Valdez BJ, Matsuba C (2024). Biological effects of intraoperative radiation therapy: histopathological changes and immunomodulation in breast cancer patients. Front Immunol.

[18] Torabinejad S, Soleymanifard S, Sayyah S, Behnam Rasouli F (2023). High-dose irradiation stimulated breast tumor microenvironment to enhance tumor cell growth and decrease tumor cell motility. J Biomed Phys Eng.

[19] Nafissi N, Mohammadlou M, Akbari ME (2022). The impact of intraoperative radiotherapy on breast cancer: focus on the levels of angiogenic factors. World J Surg Oncol.

[20] Pan L, Huang Z, Zhan Q, Zhang X, Tang W, Zheng W (2022). Long term effect of INTRABEAM single irradiation on the expression of miRNAs inMCF-7 cells. J Radiat Res Appl Sci.

[21] Wuhrer A, Uhlig S, Tuschy B, Berlit S, Sperk E, Bieback K, Sütterlin M (2021). Wound fluid from breast cancer patients undergoing intraoperative radiotherapy exhibits an altered cytokine profile and impairs mesenchymal stromal cell function. Cancers (Basel).

[22] Kulcenty K, Piotrowski I, Rucinski M, Wroblewska JP, Jopek K, Murawa D, Suchorska WM (2020). Surgical wound fluids from patients with breast cancer reveal similarities in the biological response induced by intraoperative radiation therapy and the radiation-induced bystander effect-transcriptomic approach. Int J Mol Sci.

[23] Kulcenty K, Piotrowski I, Wróblewska JP, Wasiewicz J, Suchorska AW (2019). The composition of surgical wound fluids from breast cancer patients is affected by intraoperative radiotherapy treatment and depends on the molecular subtype of breast cancer. Cancers (Basel).

[24] Kulcenty KI, Piotrowski I, Zaleska K, Murawa D, Suchorska WM (2018). Wound fluids collected from patients after IORT treatment activates extrinsic apoptotic pathway in MCF7 breast cancer cell line. Ginekol Pol.

[25] Linares I, Berenguer MA, Martínez E (2019). INTRABEAM: precision hypo-fractionated radiotherapy with a systemic immune response. Radiother Oncol.

[26] Kulcenty K, Piotrowski I, Zaleska K, Wichtowski M, Wróblewska J, Murawa D, Suchorska WM (2019). Wound fluids collected postoperatively from patients with breast cancer induce epithelial to mesenchymal transition but intraoperative radiotherapy impairs this effect by activating the radiation-induced bystander effect. Sci Rep.

[27] Zaleska K, Przybyła A, Kulcenty K, Wichtowski M, Mackiewicz A, Suchorska W, Murawa D (2017). Wound fluids affect miR-21, miR-155 and miR-221 expression in breast cancer cell lines, and this effect is partially abrogated by intraoperative radiation therapy treatment. Oncol Lett.

[28] Scherer SD, Bauer J, Schmaus A (2016). TGF-β1 is present at high levels in wound fluid from breast cancer patients immediately post-surgery, and is not increased by intraoperative radiation therapy (IORT). PLoS One.

[29] Fabris L, Berton S, Citron F (2016). Radiotherapy-induced miR-223 prevents relapse of breast cancer by targeting the EGF pathway. Oncogene.

[30] Veldwijk MR, Neumaier C, Gerhardt A, Giordano FA, Sütterlin M, Herskind C, Wenz F (2015). Comparison of the proliferative and clonogenic growth capacity of wound fluid from breast cancer patients treated with and without intraoperative radiotherapy. Transl Cancer Res.

[31] Park KU, Showalter SL, Dirbas FM (2024). Surgical perspectives on the updated ASTRO guideline on partial breast irradiation for breast cancer. Ann Surg Oncol.

[32] Vaidya JS, Baum M, Tobias JS (2011). Long-term results of targeted intraoperative radiotherapy (Targit) boost during breast-conserving surgery. Int J Radiat Oncol Biol Phys.

[33] Vinante L, Vaidya JS, Caroli A (2024). Real world clinical outcomes from targeted intraoperative radiotherapy (TARGIT-IORT) during lumpectomy for breast cancer: data from a large cohort at a national cancer institute. Front Oncol.

[34] Vaidya JS, Bulsara M, Wenz F (2023). The TARGIT-A randomized trial: TARGIT-IORT versus whole breast radiation therapy: long-term local control and survival. Int J Radiat Oncol Biol Phys.

[35] Dai D, Li X, Zhuang H (2025). Landscape of the peripheral immune response induced by intraoperative radiotherapy combined with surgery in early breast cancer patients. Adv Sci (Weinh).

[36] Coombs NJ, Coombs JM, Vaidya UJ (2016). Environmental and social benefits of the targeted intraoperative radiotherapy for breast cancer: data from UK TARGIT-A trial centres and two UK NHS hospitals offering TARGIT IORT. BMJ Open.

[37] Bhimani F, McEvoy M, Chen Y (2024). Case report: IORT as an alternative treatment option for breast cancer patients with difficulty staying still. Front Oncol.

[38] Corica T, Nowak AK, Saunders CM (2016). Cosmesis and breast-related quality of life outcomes after intraoperative radiation therapy for early breast cancer: a substudy of the TARGIT-A trial. Int J Radiat Oncol Biol Phys.

[39] Yang Y, Hou X, Kong S (2023). Intraoperative radiotherapy in breast cancer: alterations to the tumor microenvironment and subsequent biological outcomes (review). Mol Med Rep.

[40] Brunt AM, Haviland JS, Wheatley DA (2023). One versus three weeks hypofractionated whole breast radiotherapy for early breast cancer treatment: the FAST-Forward phase III RCT. Health Technol Assess.

[41] Vaidya A, Vaidya P, Both B, Brew-Graves C, Bulsara M, Vaidya JS (2017). Health economics of targeted intraoperative radiotherapy (TARGIT-IORT) for early breast cancer: a cost-effectiveness analysis in the United Kingdom. BMJ Open.

[42] Pohlers D, Brenmoehl J, Löffler I (2009). TGF-beta and fibrosis in different organs - molecular pathway imprints. Biochim Biophys Acta.

[43] Anscher MS (2010). Targeting the TGF-beta1 pathway to prevent normal tissue injury after cancer therapy. Oncologist.

[44] Redis RS, Calin S, Yang Y, You MJ, Calin GA (2012). Cell-to-cell miRNA transfer: from body homeostasis to therapy. Pharmacol Ther.

